# Discovering Hidden Mental States in Open Multi-Agent Systems by Leveraging Multi-Protocol Regularities with Machine Learning

**DOI:** 10.3390/s20185198

**Published:** 2020-09-12

**Authors:** Emilio Serrano, Javier Bajo

**Affiliations:** Ontology Engineering Group, Department of Artificial Intelligence, Universidad Politécnica de Madrid, 28660 Madrid, Spain; jbajo@fi.upm.es

**Keywords:** open multi-agent system, smart city, agent communication languages, agent-oriented software engineering

## Abstract

The agent paradigm and multi-agent systems are a perfect match for the design of smart cities because of some of their essential features such as decentralization, openness, and heterogeneity. However, these major advantages also come at a great cost. Since agents’ mental states are hidden when the implementation is not known and available, intelligent services of smart cities cannot leverage information from them. We contribute with a proposal for the analysis and prediction of hidden agents’ mental states in a multi-agent system using machine learning methods that learn from past agents’ interactions. The approach employs agent communication languages, which is a core property of these multi-agent systems, to infer theories and models about agents’ mental states that are not accessible in an open system. These mental state models can be used on their own or combined to build protocol models, allowing agents (and their developers) to predict future agents’ behavior for various tasks such as testing and debugging them or making communications more efficient, which is essential in an ambient intelligence environment. This paper’s main contribution is to explore the problem of building these agents’ mental state models not from one, but from several interaction protocols, even when the protocols could have different purposes and provide distinct ambient intelligence services.

## 1. Introduction

Smart cities are technologically based on the combination of several socio-technical innovations such as: the Internet of Things (IoT), mobile Internet access, smartphones, data analytics, open data initiatives, and sharing economy models, among others [1]. This allows these cities to manage assets and resources efficiently by services enhanced with intelligence such as: traffic management, hospitals, transportation systems, power and water plants, waste management, etcetera.

The open and heterogeneous nature of multi-agent systems (MASs) addresses naturally the dynamism and scalability problems of smart cities and ambient intelligence [2]. High-level interaction protocols and communications are a cornerstone of MASs, which are capable of establishing conversation by following these protocols, sending and receiving messages, or sharing vocabularies.

However, how do we leverage information from an open MAS to provide citizens with intelligent services? MAS platforms and frameworks usually allow developers to analyze agents’ mental states and interactions among agents for testing, debugging, and verification purposes [3]. Regarding the study of agents’ mental states, these tools tend to assume that the agents’ implementation is available. This is not the case in an open MAS for a smart city or large-scale ambient intelligence systems where the use of MASs is motivated, among others, because of agents’ capacity of migration from one to another platform at run-time. Moreover, these agents can be designed and implemented by different developers and operators, which may want to use proprietary code. Concerning interaction analysis, these tools usually study fixed elements in messages and protocols such as the performative or the sender without analyzing the message semantics and content. This hinders smart city users, developers, and agents from performing a number of interesting tasks beyond using a black-box service such as verifying or validating agents or analyzing their trustworthiness. We address these drawbacks and limitations in the specialized literature by leveraging semantic information in agents’ interaction protocols to build mental state models and protocol models with machine learning methods.

Let us consider the FIPAcontract-net protocol [4] to illustrate the utility of studying and discovering agents’ mental states. In this protocol, one agent (the Initiator) takes the role of manager, which wishes to have some task performed by one or more other agents (the Participants) and further wishes to optimize a function that characterizes the task [4]. This protocol can be used in several services of a smart city as transport renting or accommodation finders. The specialized literature [3] emphasizes syntactic analysis of the conversations such as: statistics about how many conversations an agent has started; errors in the order of the messages specified in the protocol; participants whose proposal is rejected, etcetera. However, the specific semantics of messages, which can be made explicit in the protocol definition via semantic annotations, contains more interesting information. For instance, the Initiator agent (or its developer) may want to know what is a Participant’s mental state or decision rules to propose or refuse a specific type of act. Of course, this information is hidden if the Participant agents’ implementation is unknown by the Initiator, which is the case in a smart city. Nonetheless, as in human interactions, the Initiator agent can get insights into a Participant’s behavior after participating in several conversations with it. Hopefully, the Initiator can generalize a theory about this participant’s decision-making mechanisms and act intelligently based on this theory, for instance not starting conversations with a participant who seems unwilling to conduct a specific kind of task. In this example, the Initiator would reduce the load of the communications network, which is essential in IoT, by leveraging a theory about the Participant’s hidden mental state.

These theories about agents’ mental states try to predict logical constraints and decision rules, which are typically attached to the specification of interaction protocols. Machine learning methods can leverage regularities in previously observed interactions to build a model capable of getting insights into these hidden mental states used by agents for decision-making. Then, these machine learning models can be used in several manners, such as: predicting agents’ future behavior for improving negotiations or reducing the communications; verifying or validating agents whose implementation is not available by checking if the expected behavior meets machine learning models’ predictions; or analyzing the trustworthiness of agents in a multi-agent system.

The main contribution of this paper over some previous works [5,6,7] is the specification of several approaches to leverage information from different protocols to build hidden mental state models and protocol models combining them. Returning to the example of the FIPA contract-net protocol, when the initiatorasks a participant to undertake some task *T*, at some point in the execution, the participant considers if it is interested or able to do *T*. This evaluation depends more on the task, the semantics in the protocol, than on the protocol itself. As a matter of fact, this same evaluation would be present if the protocol used was FIPA request [8]. Therefore, this paper claims that information from executions of FIPA request could be used to gain insights into the behavior of agents for FIPA contract-net. In other words, when several protocols share semantics properties, the behavior of agents when they interact by one of them can be extrapolated from the interactions by the remaining protocols. For instance, the dates negotiated for an accommodation service in a smart city could be used to negotiate available transport.

This paper shows experimental results in a car renting multi-agent system to illustrate the potential of our approach. Past agents’ interactions are used: to feed general machine learning algorithms; to learn a specific hidden mental state in an agent; to predict negotiation outcomes; to study incoherent behaviors and changes in agents’ preferences; and to use information from other protocols, an online game in the case study, to improve the learned models for the car renting system.

The paper outline is the following. Section 2 revises the related works. Section 3 introduces the formal framework of our proposal. Then, Section 4 discusses different approaches to build hidden mental state models from different interaction protocols. Section 5 gives empirical results obtained in a case study. Finally, Section 6 concludes.

## 2. Related Work

In a recent systematic review about agent systems’ verification, Bakar and Selamat [3] analyzed 231 research works and concluded that only 25% of these approaches are suitable for run-time analysis. Model checking has been the main method for agents’ verification (142 works). The second main line has been debugging or testing techniques performed during the development phase to detect faults and agents’ property violations (40 works). However, only 21 works were found to check the satisfactions of agent properties at run-time. As stated in the Introduction, this is the main scenario in the use of multi-agent system to design smart cities, where agents from different users and manufacturers have to coexist in an artificial society without being able to impose a design method. Our approach considers a trace with all messages exchanged and constraints with semantic annotations as training data for machine learning models that, after being trained, are used for run-time analysis.

Most methods and tools for the analysis of run-time multi-agent executions are meant to be used for testing and verification purposes when the agents’ implementation is open and available. In this manner, the JADEXagent platform counts with tools [9] capable of verifying the events and messages declared and transforming them into a graph. In the Agent Factory Agent Programming Language (AFAPL), the inspector tool [10] allows developers to inspect agents’ internal states and to monitor agents’ performance. The INGENIAS [11,12] platform integrates visual inspection tools for testing and debugging agents’ mental states. These and other approaches such as the Tracer Tool [13] are not suitable for a multi-agent system where agents’ code can be proprietary and not disclosed. However, these are very useful and valuable tools for multi-agent system developers in their code.

Many agent development frameworks also offer graphical tools for the visual inspection of interactions (e.g., [14]). These are usually built on a three-step procedure: (1) defining protocols that specify the interaction between agents, (2) automatically testing that these protocols were correctly performed, and (3) locating the errors found using some sort of visualization. Padgham et al. [15] used a translation from AUMLprotocols to Petri-nets, which can be used by the debugger to monitor conversations and throw error messages when protocols are not followed correctly. The typical errors that can be identified in multi-agent systems using this approach [15] are: uninitialized agent, failure to send, the wrong recipient, message sent multiple times, and wrong message sent. Other approaches use extensions of the propositional dynamic logic [16], statecharts [17], or Dooley graphs [18]. Chopra et al. [19] formalized the semantic relationship between agents and protocols, which allows the human designer to verify if a protocol supports particular agent goals and whether the agent’s specification supports the satisfaction of particular commitments required by a protocol.

In contrast to the run-time analysis of multi-agent systems, another main approach to MAS analysis considers only static property agents. In this vein, MABLE [20] is an imperative programming language for the design and automatic verification of MASs. MABLE allows developers to design agents’ mental states following the beliefs, desires, and intentions paradigm. By using linear temporal logic, MASs implemented in MABLE can use the spin model checker to be automatically verified. More recently, the MCAPLframework by Dennis et al. [21,22] provided a suite of tools for building interpreters for agent programming languages and verifying the correctness of programs running in these interpreters using the model checking technique. Again, this approach is not valid when the agents’ code is not available. Besides, these works focus on the verification of a single agent’s behavior or reasoning according to the (single) agent’s beliefs and goals, omitting the verification of the interaction of multiple agents.

Besides MAS analysis works, there are similarities between ontology matching [23,24,25] proposals and the method presented in this paper. Among others, both research lines are based on considering the semantics of terms and concepts appearing in previous agents’ conversations and looking for relationships between them. On the other hand, in the ontology alignment literature, the purpose is not inferring models of agents’ hidden mental states, but resolving conflicts between ontologies used by these agents. Ancona et al. [26,27] also explored the semantic definition of the protocols by parametric trace expressions for run-time verification. As in our approach, the authors pointed out that the correctness of protocol interactions depends on the specific data, and that, in general, cannot be predicted statically. However, the proposed automatic verification cannot detect MAS’s emergent behaviors like the use of machine learning methods in our proposal.

As explained in this section, the main limitation in the existing works is that the semantics of messages exchanged between agents is not contemplated for the analysis of their behavior. As pointed out by Savaglio et al. [28] in a recent review about the use of MAS in the IoT, software agents have evolved in “non-semantic directions”. Moreover, there are very few works that attempt to build compact models of agents’ mental states when their code is not available [5,6,7]. Last, but not least, combining information from different interaction protocols to discover agents’ hidden mental states is a novel problem introduced in this paper. We consider the methods presented here as an unexplored opportunity for providing intelligent services in smart cities with new methods from leveraging information from unknown agents.

## 3. Formal Approach for Discovering Hidden Mental States in Multi-Agent Systems

In this section, we specify a formal framework for defining protocols with semantic annotations and how to leverage these annotations for building hidden mental state models using machine learning methods. This framework only covers the minimum amount of requisites to allow our approach to be used. Moreover, the extension of specific MAS platforms to cover these requirements is outside of the scope of this paper.

### 3.1. Defining a Semantically Annotated Protocol

A MAS interaction protocol can be formalized as a graph G=(V,E) where vertices or nodes represent messages and links or edges indicate mental states, which are evaluated by the message sender.

A node v∈V representing a message would contain at least four characteristic fields of MAS messages: a performative *q*, a sender *X*, a receiver (or a list of them) *Y*, and the message content *Z*. Therefore, each node/message can be labeled with a semantic annotation m(v)=q(X,Y,Z).

A link representing a mental state could be any predicate ϕ∈L of a logical language L. However, for the sake of simplicity, our semantic protocol definition labels each link with a conjunction of *n* constraints or decision-making functions (or better said, predicates, since they are evaluated with a truth value). These links can be labeled with $c\left(e\right)=\{c_{1}\left(t_{1},\;\ldots ,\;t_{k_{1}}\right),\;\ldots ,\;c_{n}\left(t_{1},\;\ldots t_{k_{n}}\right)\}$ where each of the constraints $c_{i}$ that composes the mental state has arity $k_{i}$ and arguments $t_{j}$ indicating terms that can be replaced at run-time by a constant, functions, or variables. As in Prolog and other logic programming languages, all variables are considered universally quantified. The outcoming links or messages from a node or mental state have distinct performatives, i.e., for all $\left(v,v^{\prime }\right),\left(v,v^{\prime \prime }\right)\in E$, $\left(m\left(v^{\prime }\right)=q\left(\ldots \right)\wedge m\left(v^{\prime \prime }\right)=q\left(\ldots \right)\right)\Rightarrow v^{\prime }=v^{\prime \prime }$.

Note that the protocol only specifies the header with the name and arguments of the constraints composing the agents’ mental states, the code and its evaluation considering these arguments and other agents’ beliefs and desires stored in each agent. As explained below, this allows our approach to discover mental state (machine learning) models. On the other hand, the exact mental states may not be leveraged from these machine learning models following our approach, which essentially considers a trace with all messages exchanged and constraints evaluated as training data for these models. Another disadvantage of our approach is that this protocol definition moves complexity from the agents to the interaction protocol, which may not be desirable in a layered architecture. Note also that other modeling approaches for defining interaction protocols may be used as long as the messages and constraints are semantically labeled, e.g., representing messages as edges instead of nodes in the graphs, or using enhanced UMLor AUML diagrams.

**Figure 1:**
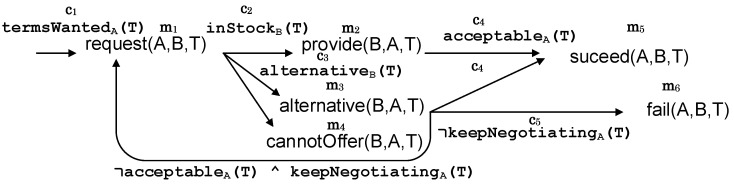
A negotiation protocol model where an initiator *A* asks for a product with some terms *T* from a provider *B*. *B* replies based on the availability of an item with the requested terms. When *T* cannot be satisfied by *B*, *A* and *B* can negotiate new terms for the product. Several predicates representing participants’ mental states are evaluated during this negotiation: keepNegotiating, acceptable, and alternative. Subscript *A* or *B* in link labels represent the agent that has to evaluate the predicate. Additional (redundant) shorthand notation $c_{i}$/$m_{j}$ is introduced indicating different constraints and messages, respectively.

The result is that when two or more agents interact using a protocol defined with these requirements, the produced message sequence corresponds to one path π in *G*. Figure 1 illustrates this generic format for defining protocols semantically annotated for illustration purposes.

The semantics of a protocol *G* can be defined considering the pairs 〈π,θ〉 containing the path and variable substitution that any message sequence *m* corresponds to in the protocol *G*, the context of *m* the expression being $C\left(G\left(m\right)\right)={⋀}_{i=1}^{n-1}c\left(e_{i}\right)\theta$ where $m=\langle m_{1},\;\ldots ,\;m_{n}\rangle$, G(m)=〈π,θ〉, and *c* are constraints or mental states associated with each link in the path π. More importantly, the conjunction of mental states described in nodes or message sequence *m* of a path π in the protocol *G* is logically true at the time of the interaction.

The problem addressed in this paper is how to use these paths with ground terms, pairs 〈π,θ〉, to allow machine learning to learn hidden mental state models of all participants and protocol models capable of predicting the conversations’ outcome. More specifically, how do we build these models when several protocols (or graphs $G_{1},G_{2},\ldots ,G_{n}$) are considered?

### 3.2. Obtaining Hidden Mental State Models with Machine Learning

The basic method for applying machine learning methods to agents’ conversations following the formal protocols defined in the previous section is the following. Given a protocol model *G* and a message sequence *m* from a past conversation following *G*, the sequence can be translated into a pair G(m)=〈π,θ〉. These substitution-annotated paths are preprocessed as discussed in Section 3.3 to obtain a training dataset *D*. Then, an inductive machine learning algorithm L:D→H can map any concrete dataset D⊆D, where D is the set of all possible observations, to a learning hypothesis h∈H, which constitutes a hidden mental state model for the protocol *G*.

Our approach assumes that only allowed message sequences occur, but some verification approaches revised in Section 2 can be used to ensure this. Moreover, our approach could be modified on the fly to accommodate unexpected messages by adding constraint-free links and message nodes. Finally, the learning data *D* can be augmented by the logical context of the data samples including inferable data from the logical formula C(G(m)).

**Figure 2:**
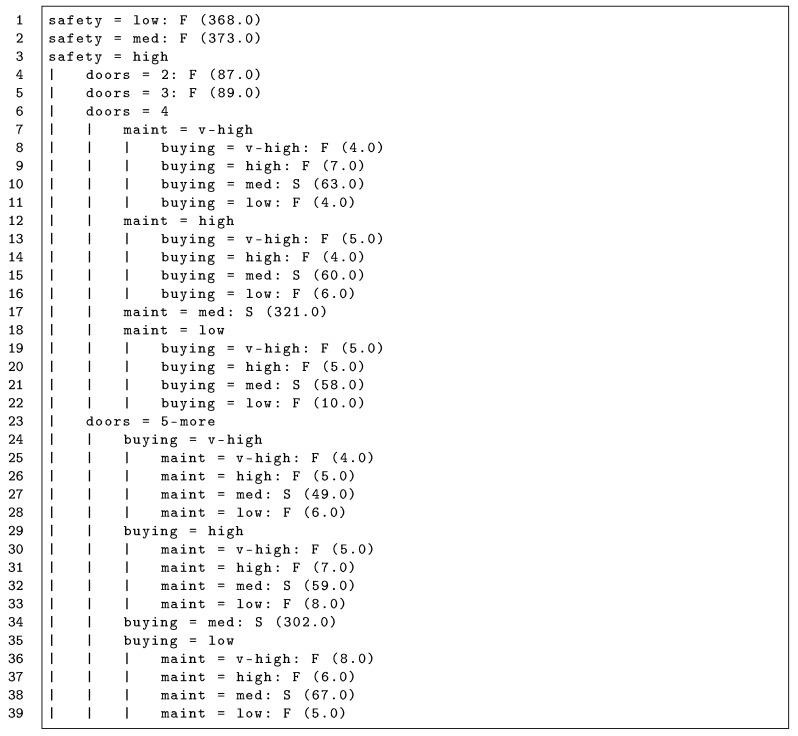
Hidden mental state model of the $acceptable_{A}$ constraint, obtained using a decision tree algorithm (J48) with a dataset gathering 2000 conversations, which follow the protocol defined in Figure 1. The notation “a = v : T/F” indicates that “if the term *a* has value *v*, the target predicate has a value of true or false”. Every leaf includes the number of instances classified under a certain branch in parentheses.

As explained in the following section, the machine learning task involved in obtaining these hidden mental state models is a classification where the class labels reflect the outcome of a protocol for protocol models, or constraints occurring within the protocol for the hidden mental state model. Figure 2 illustrates a hidden mental state model for the the constraint $acceptable_{A}$ of the protocol described in Figure 1 when negotiating a car rental. This specific model uses a decision tree as a machine learning method, but any other algorithm suitable for classification could be employed.

### 3.3. Obtaining Training Data for Hidden Mental State Models

This section describes the additional design decision to be made before standard machine learning methods can be used. This involves essentially the transformation of message sequences or graph paths into tabular data composed of same-length vectors. As mentioned in the Future Work section, recurrent neural networks that deal with sequences or graph neural networks [29] that can learn from graphs represent possible alternatives to be explored.

The first major decision to be made is how to cope with multiple agents in conversations. The purpose of our approach is to infer the definitions of constraints or hidden mental states that are specific to an agent or a group of agents. To filter the data, assume an assignment σ:Var→Ag where Ag is the set of agent names and Var is the set of all variables occurring as sender or receiver variables in the graph nodes. Then, for any agent a∈Ag, $V_{\sigma }\left(a\right)$ are the nodes that correspond to messages sent by agent *a* under role assignment σ, and $E_{\sigma }\left(a\right)$ are the incoming links to those nodes/messages. This can be formally expressed as $E_{\sigma }\left(a\right)=\left\{\left(v,v^{\prime }\right)\in E|v^{\prime }\in V_{\sigma }\left(a\right)\right\}$. These notions can be generalized to $V_{\sigma }\left(A\right)/E_{\sigma }\left(A\right)$ for A⊆Ag by taking the union over the respective sets of agents.

A second decision is how to deal with the paths of different lengths when building the training data tuples since the messages, constraints, and variables contained in them can differ. A simple solution is to “pad” these tuples with “unknown” values for all messages and mental state variables not occurring in them. Therefore, the length of the tuple will depend on the longest path. A similar “padding” strategy is used in several deep learning methods such as convolutional neural networks or the BERT(pre-training of deep bidirectional transformers for language understanding) model [30]. Another alternative would be creating different datasets for different paths π in the protocol graph *G*. Finally, different paths can be merged into a single set introducing an artificial label with the conversation result, e.g., success=true.

Thirdly, the results of the hidden mental states in the paths (but not the terms of these) must be removed. Otherwise, machine learning methods would learn obvious relations between these mental states and the conversation’s overall outcome, e.g., “if not continuing to negotiate, then the conversation fails” in the protocol example given in Figure 1.

Fourthly, the existence of a loop in a protocol means that variables occurring in a hidden state or message can have several constants as ground instantiations in the same conversation $\left\{g_{1},g_{2},\ldots ,g_{n}\right\}$, where *n* is the number of iterations in the loop. This is a multi-instance learning problem, i.e., learning where each example in the data comprises several different instances [31]. A simple strategy is to consider only the first/last ground term $g_{1}/g_{n}$ or other aggregate functions such as the average, minimum, or maximum of the ground terms. More advanced multi-instance methods can also be considered [32].

## 4. Leveraging Multi-Protocol Regularities for Hidden Mental State Models

One possibility for the hidden mental state models presented in this paper that has not been previously explored in the literature is to gain knowledge about agents’ behavior in the performance of a protocol by using information from a second protocol different, or potentially, from a complete set of different protocols.

In this section, a first protocol under study $p_{1}$ with a possible execution path $\pi_{1}$ is considered. Then, the hidden mental state models learned for this protocol are enhanced by data from interactions in a second or auxiliary protocol $p_{2}$ whose possible execution path is $\pi_{2}$.

### 4.1. Combining Complete Paths

Let us call $p_{1}$ the protocol under analysis and $p_{2}$ the “auxiliary” protocol. The basic method to extend the tuples of the training data, which allow a protocol model for $p_{1}$ to be built with $p_{2}$ data, is to find paths in $p_{2}$ that include all the constraints that a complete path in $p_{1}$ presents (where a complete path is a path from the beginning of the protocol to a final message); in other words, finding paths of $p_{1}$ whose constraints, ordered or not, also are present in paths of $p_{2}$, with other constraints or not. For example, if we consider as $p_{1}$ the protocol shown in Figure 1 to negotiate the sale of a product, the shortest complete path this protocol has is the following:

(1) $$\pi_{1}=\overset{termsWanted}{\to }request\overset{\neg inStock\wedge \neg alternative}{\to }cannotOffer$$

A seller agent implementing this protocol can also implement, for example, a protocol to offer advertising about products offered or a protocol for auctions. In this possible second protocol, $p_{2}$, the path $\pi_{1}$ is feasible. In this case, sellers may, and indeed human sellers do, use the information gained through advertising protocols to improve their sales negotiation protocols. In this proposal, and once the agents have implemented a learning strategy for a specific protocol as explained in Section 3.3, checking for common constraints in protocols to combine their tuples is completely automatic.

### 4.2. Combining Partial Paths

This first approach for combining protocols, although automatic and innovative, is also very restrictive. Of course, designers can implement protocols aiming at maximizing the reusability of paths for discovering hidden mental states. However, in practice, finding protocols with disparate purposes sharing long sequences of mental processes is difficult. To solve this, a second combination approach is presented here.

As explained in Section 3.2, the class of a hidden mental state model does not have to be the overall output of the protocol. This class can also be an unknown mental process that is implemented by another agent whose behavior is under analysis. Therefore, all the constraints of complete paths of $p_{1}$ do not have to occur in $p_{2}$, but only the constraints from the beginning of the protocol in $p_{1}$ to the hidden mental state that is used as a class. Therefore, at the cost of not being able to articulate a theory for the protocol in general, this approach allows developers to reuse larger amounts of information than the first. Returning to the example of the protocol in Figure 1, if the goal is to construct a hidden mental state model explaining the behavior of the constraint $c_{3}=alternative$, tuples from executions of a second protocol can be included if those executions include the constraints in:

(2) $$\pi_{2}=\overset{termsWanted}{\to }request\overset{\neg inStock\wedge alternative}{\to }$$

This is possible because the information beyond that sequence, and that makes the protocol $p_{1}$ end in different ways, is irrelevant to study the generation of alternatives to out-of-stock orders by the seller.

### 4.3. Finding Logical Consequence in Mental States of Different Protocols

While the second approach is much less restrictive than the first, finding sequences encompassing all constraints of paths of the protocol to be analyzed in different protocols can be difficult. A third approach that this paper proposes is to look for logical consequence relationships between the constraints of $p_{2}$ and $p_{1}$ to replace the results of the constraints in the latter by the results of the constraints in the former. For example, consider a constraint $c_{1}=isExpensive\left(T\right)$ in a protocol $p_{1}$, that determines whether a product is expensive based on the terms, and a protocol $p_{2}$ that does not use $c_{1}$, but $c_{2}=isLuxurious\left(T\right)$, which determines whether a product is luxurious. Assuming isLuxurious⇒isExpensive, then every interpretation that makes isLuxurious true also makes isExpensive true.

Therefore, we can add tuples with the parameters and results of $c_{2}$ in $p_{2}$, for those cases where $c_{2}$ is evaluated as true, to study $p_{1}$. More generally, when the auxiliary protocol constraints are evaluated as true, the constraints in the protocol under analysis also have to be true. This simple example can be generalized to more complex arguments where several constraints are considered in the antecedent of the logical consequence, i.e., $\left\{c_{2},c_{3},\ldots ,c_{n}\right\}\Rightarrow c_{1}$.

As in the strategies to form the training data explained in Section 3.3, smart city designers can find these relationships for a set of protocols given in advance. Then, the agents can combine information from different protocols automatically. Moreover, the use of ontologies allows intelligent services to discover these logical consequences automatically [33]. For instance, the basic Resource Description Framework (RDF) schema [34] can specify that Luxurious is a *subClassOf*Expensive, which formalizes an if-then sentence without requiring more expressive logic such as the Web Ontology Language (OWL) [35] or horn-like rules such as the Semantic Web Rule Language (SWRL) [36].

### 4.4. Using Hidden Mental State Models to Pseudo-Label Unknown Constraints

Finally, a fourth approach consists of using hidden mental state models of constraints to complete tuples in $p_{2}$ to be used as tuples for $p_{1}$ when the former protocol, despite not having a constraint needed, has the parameters for this mental process. While this approach will not be treated in the experimental results section, the idea is perfectly feasible since, as proven in Section 5.3, a model of a specific constraint can not only be very accurate, but also exact. Therefore, the predictions of this model can be used to complete the necessary information in a path of $p_{2}$. For example, consider the protocol of Figure 1 as $p_{1}$, the path $\pi_{1}$ reflected in the expression (1), and a $p_{2}$ protocol with the following possible path $\pi_{2}$:

(3) $$\pi_{2}=\overset{termsWanted}{\to }query\overset{\neg alternative}{\to }reject$$

With the above methods, $p_{1}$ cannot include executions of $\pi_{2}$ because the constraint inStock is only in $\pi_{1}$. However, if there is enough information about the executions of $p_{1}$ to construct a very accurate model of inStock, agents can use this model to predict the output of inStock taking as parameters the data from the executions of $\pi_{2}$. Then, this execution of $\pi_{2}$ can be used as a tuple representing an execution of $\pi_{1}$ provided: (1) the output predicted by the model for inStock is false, (2) termsWanted=true in $\pi_{2}$, and alternative=false in $\pi_{2}$. This strategy is reminiscent of the pseudo-labeling method, which is one of the simplest approaches of semi-supervised learning for deep neural networks [37].

Note that none of the strategies discussed in this section require similarities in messages between different protocols, but only the terms used in them. Therefore, our approach to learning hidden mental state models and protocol models focuses on semantics, unlike most of the proposals revised in Section 2.

## 5. Case Study

This section describes a multi-agent system in a car renting domain to illustrate how to discover hidden mental states by leveraging multi-protocol regularities with machine learning. The agents in the system interact using the protocol shown in Figure 1 and a second protocol described in Figure 5.

Section 5.1 starts giving a description of this case study, and then, Section 5.2 explains the strategies used to gather a dataset to build hidden mental state models. Section 5.3 describes how to build these models to explain an unknown mental process. Beyond building models for a single hidden mental state in an interaction protocol, Section 5.4 illustrates the use of the methods presented in this paper to construct a model capable of explaining a whole protocol. Section 5.5 details a practical application of these models, which is studying the coherence of agents’ behavior. Finally, Section 5.6 combines two different protocols to generate a single protocol model.

### 5.1. Description of the Multi-Agent System

The MAS used in this section negotiates cars with the terms and features described in a public database for car evaluation [38] where cars are described by technical characteristics and prices. The dataset contains 1728 instances and six attributes. More specifically, car characteristics are given as a tuple T=(doors,persons,lug_boot,safety,buying,maint). The possible values for these attributes are the following:

- *buying*: vhigh, high, med, low.
- *maint*: vhigh, high, med, low.
- *doors*: 2, 3, 4, 5, more.
- *persons*: 2, 4, more.
- *lug_boot*: small, med, big.
- *safety*: low, med, high.

The data do not contain missing values and are associated with classification tasks, the possible class values being: unacc, acc, good, or vgood.

Agents in the system follow the negotiation protocol detailed in Figure 1. In this manner, a customer with the role *A* can request offers from a car renting agent with the role *B* giving the wanted terms for the car *T* (such as number of doors, capacity, the size of luggage boot, and estimated safety). This case study was implemented using the MASON Multiagent Simulation Toolkit (https://cs.gmu.edu/~eclab/projects/mason/).

In our experiments, ten customer or renter agents are defined ($C_{i}$, where 1≤i≤10). These agents have five different mental states associated with them, which correspond to different preferences regarding *T* that determine what offers they will accept. $C_{i}:={\mathrm{MS}}_{i\;mod\;5}$, meaning that: agents $C_{1}$ and $C_{6}$ have mental state ${\mathrm{MS}}_{1}$, $C_{2}$ and $C_{7}$ have mental state ${\mathrm{MS}}_{2}$, and so on. For example, the mental state indicating $C_{1}$ preferences in the MAS is the following:

$$\begin{array}{cc} {\mathrm{MS}}_{1}\left(T\right)\Leftrightarrow & (\mathrm{doors}=4\wedge \mathrm{safety}=\mathrm{high}\wedge \mathrm{maint}=\mathrm{med})\vee \\ & (\mathrm{doors}=4\wedge \mathrm{safety}=\mathrm{high}\wedge \mathrm{buying}=\mathrm{med})\vee \\ & (\mathrm{doors}=5-\mathrm{more}\wedge \mathrm{safety}=\mathrm{high}\wedge \mathrm{maint}=\mathrm{med})\vee \\ & \left(\mathrm{doors}=5-\mathrm{more}\wedge \mathrm{safety}=\mathrm{high}\wedge \mathrm{buying}=\mathrm{med}\right) \end{array}$$

The case study assumes that a single seller or lessor agent *S* is studying the system’s evolution from its local point of view to predict the different outcomes of its interactions based on perceived regularities regarding the observed behavior of the customers or renters.

### 5.2. Building the Training Data for Discovering Hidden Mental States

This section describes the strategies to build a training dataset from sequences of message exchanges in the case study by using some of the methods detailed in Section 3.3.

As explained above, a seller or lessor agent with name *S* (B=S in the protocol specification in Figure 1) is performing the analysis to obtain knowledge about the other agents’ hidden mental states. Therefore, the learning input is restricted to the mental states in the customer agents, i.e., $V_{c}\left(A\right)$ and $E_{c}\left(A\right)$, *c* being the customer role in the protocol. All attributes contained in the “terms” descriptions *T* are gathered including an unknown value for those not mentioned in a given execution trace. To address loops, only the last value of every term is gathered for the training data. Finally, an artificial variable outcome∈{S,F,N} is introduced to indicate a conversation with a successful completion ($m_{11}$ is the last message in the protocol of Figure 1), failure (conversation ends with $m_{4}$ or $m_{6}$), and neutral for the remaining paths.

Even thought evaluating the best machine learning algorithm for the analysis of opaque mental states is out of the scope of this paper, five open-source implementations are employed [39]. These include a decision tree algorithm (J48), a rule induction method (NNge), a Bayesian network classifier (BayesNet), a feed-forward artificial neural network (multilayer perceptron), and a clustering-based classification (SimpleKMeans). This selection includes some of the major learning paradigms in machine learning. Moreover, this mix of white-box and black-box classifiers will prove in our experimental results that sometimes, white-box models can perform equally well as black-box models, while the former are greatly superior in supporting other extremely significant issues such as interpretability and explainability [40]. The default parameters are used for these five implementation except the number of layers in the artificial neural network (two instead of one) and the number of classes in SimpleKMeans (three instead of two to cover the three classes in the dataset, i.e., S, F, and N).

### 5.3. Learning a Hidden Mental State Model

In this experiment, the seller agent wants to learn a hidden mental state model for the predicate $c_{4}=acceptable_{A}\left(T\right)$ evaluated by a customer agent $C_{1}$. The decision tree resulting after learning from 2000 protocol executions is shown in Figure 2. The tree contains 15 nodes and summarizes the mental state of $C_{1}$. This tree can be transformed into the following logic formula (any decision tree can be transformed into a set of rules, it not being possible to transform any set of rules into a decision tree).

$$\begin{array}{cc} acceptable_{C_{1}}\left(T\right)\Leftrightarrow & (\mathrm{doors}=4\wedge \mathrm{safety}=\mathrm{high}\wedge \mathrm{maint}=\mathrm{med})\vee \\ & (\mathrm{doors}=4\wedge \mathrm{safety}=\mathrm{high}\wedge \mathrm{buying}=\mathrm{med})\vee \\ & (\mathrm{doors}=5-\mathrm{more}\wedge \mathrm{safety}=\mathrm{high}\wedge \mathrm{maint}=\mathrm{med})\vee \\ & \left(\mathrm{doors}=5-\mathrm{more}\wedge \mathrm{safety}=\mathrm{high}\wedge \mathrm{buying}=\mathrm{med}\right) \end{array}$$

The reader can check that this tree or its equivalent set of rules corresponds to the mental state implemented by the customer, see ${\mathrm{MS}}_{1}\left(T\right)$ in Section 5.1, which is opaque or hidden to the seller who has learned it.

Note that this comparison is possible because the machine learning model employed, a decision tree, is a white-box model. A white-box is a model whose inner logic, workings, and programming steps are transparent, and therefore, its decision-making process is interpretable [40]. In contrast, a black-box model, such as artificial neural networks, is a model whose inner workings are not known and are hard to interpret [40], making evaluations such as the one presented in this section impossible.

### 5.4. Protocol Outcome Prediction Leveraging Hidden Mental States’ Information

**Figure 3:**
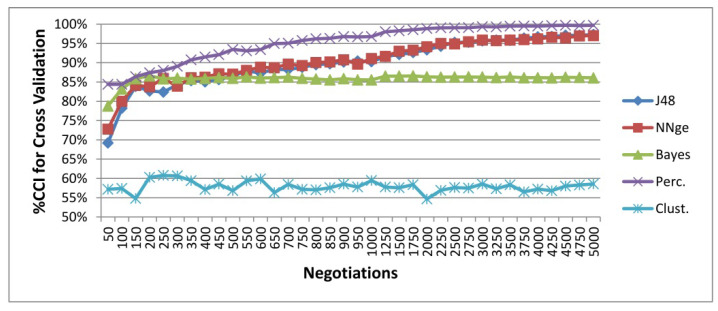
Protocol outcome prediction leveraging information from hidden mental states. The average accuracy is shown for 100 experiments with 5000 negotiations each. The learning algorithms considered include: a decision tree algorithm (J48), a rule induction method (NNge), a Bayesian network classifier (BayesNet), a feed-forward artificial neural network (multilayer perceptron), and a clustering-based classification (SimpleKMeans).

In this experiment, the seller tries to learn a model for the overall outcome of the protocol, which needs to understand not one, but all customers’ mental states, which are hidden to the seller. Figure 3 shows the average model accuracy in predicting successful or failed sales across 100 repeated experiments. The accuracy of the machine learning models is evaluated using ten-fold cross-validation across 100 experiments with 5000 negotiations each.

The results show that the models can correctly classify over 80% of all instances after considering 200 negotiations, i.e., 20 conversations for each one of the 10 customers. An exception is the use of the clustering-based classification method, which has the naive assumption that clusters will correspond to given classes. The feed-forward neural network gets the best accuracy, over 85% with only 50 negotiations and 99.70% after 5000 negotiations. This illustrates the potential of deep learning in predicting conversation outcomes, although at the cost of losing all the interpretability and explainability of the hidden mental states. J48 and NNge, which are white-box machine learning models, obtain comparable results (97.28% and 97.04%, respectively). On the other hand, J48 and NNge require more data samples, i.e., conversations observed, than the artificial neural network. Finally, the Bayes classifier gets an accuracy below 86.08%, although it reaches its maximum predictive power sooner than J48 and NNge.

Table 1 shows the time needed to build the protocol outcome models in these experiments. With 88.21 s to build a model for 5000 tuples, the feed-forward neural network is the slowest algorithm. Note that the use of ten-fold cross-validation requires training the models 11 times, this kind of validation not being typical in deep learning methods. The induction of rules only needs 1.468 s and the remaining methods below one second, the Bayes classifier being the fastest algorithm. These efficiency and scalability issues are essential in smart cities and ambient intelligence environments. The small extra accuracy achieved by the artificial neural network, 2.42%, may not justify the extra time required for training it. Moreover, the interpretability and explainability of the model can be a must in health domains such as Ambient Assisted Living (AAL) environments [41].

These experiments illustrate the potential of our approach to leverage hidden mental state information in protocol interactions to predict the final conversation outcome. Moreover, the computational cost of building these models is more than affordable for agents at run-time.

### 5.5. Studying the Coherence of Hidden Mental State Models

The trustworthiness of some renter agents can be evaluated based on an initial mental state model held by the seller, which we assume to be the decision tree model obtained from 5000 past negotiations. We use this model to classify the next 1000 negotiations performed with renters $C_{1}$, $C_{2}$, and $C_{3}$. $C_{1}$ adheres to its original preferences truthfully; $C_{2}$ picks a random new model from the set of mental states implemented for each conversation; and $C_{3}$ switches from a different mental state only once (${\mathrm{MS}}_{1}$, the mental state used by $C_{1}$ in the previous experiments).

We observe that 100% of the new runs with $C_{1}$ are correctly classified, which is in accordance with the fact that $C_{1}$ has maintained its preferences. On the other hand, we only obtain 48.75% and 43.6% correctly classified instances for $C_{2}$ and $C_{3}$, respectively. This could indicate a change in preferences, as indeed $C_{3}$ does, or the presence of contradictions in the behavior, as in the case of $C_{2}$. The seller can build a model for the latest 1000 negotiations to resolve this issue.

**Figure 4:**
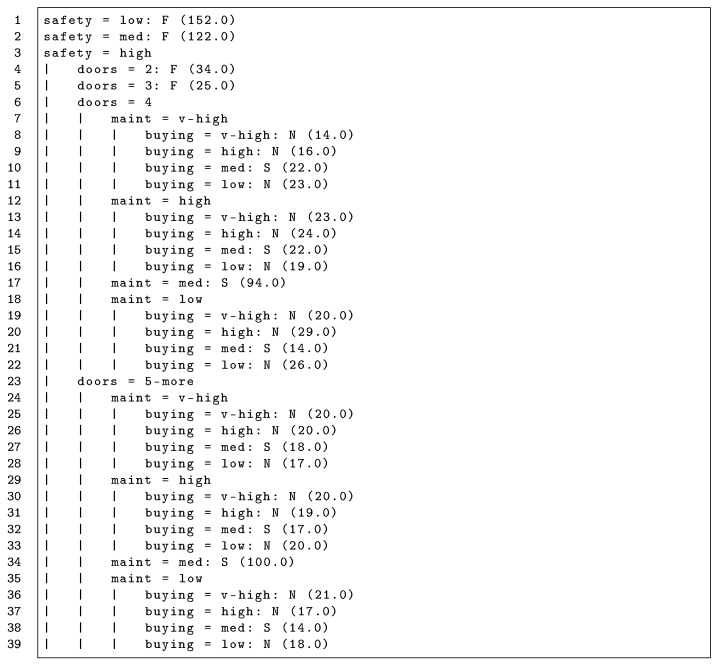
Protocol model of negotiations with $C_{3}$ after changing its preferences to $C_{1}$ ’s preferences. Model obtained using a decision tree algorithm (J48) with a dataset gathering 1000 negotiations. The notation “a = v : S/F/N” indicates that “if the term *a* has value *v*, the negotiation succeeds, fails, or is neutral”. Every leaf includes the number of instances classified under a certain branch in parentheses.

The resulting model for the communications with $C_{3}$ is shown in Figure 4. Since $C_{3}$ negotiated using $C_{1}$’s preferences (whose logic expression is shown in Section 5.1), this tree is consistent with the model obtained for the acceptable constraint of $C_{1}$, see Figure 2. Note that the trees have different labels because Figure 2 is a hidden mental state model predicting a protocol constraint or predicate (classes *T*rue or *F*alse) and Figure 4 is a protocol model predicting the conversation outcome (classes *S*uccess, *N*eutral, or *F*ailure). This model presents 100% correctly classified instances. Hence, the seller may conclude that there has been a change in preferences after studying a protocol model with only the last interactions.

On the other hand, cross-validation for the model obtained from the last 1000 interactions with agent $C_{2}$ shows 24% of instances incorrectly classified, which expresses a possibly untrustworthy or at least incoherent behavior. The seller can examine the log of negotiations and detect several contradictory conversations; see Table 2. In this table, the first and second tuples detail two negotiations regarding cars with identical terms. However, the former failed, and the latter succeeds. Three more incoherent pairs of executions are shown in the table. Hence, after a suspiciously high number of incorrect classifications with a tested model, the seller can verify that the behavior of another agent is not trustworthy by studying the semantics exchanged in the protocol.

These initial experiments only hint at the potential analyses that can be conducted regarding trust in agents and illustrate the usefulness of qualitative protocol mining in real-world scenarios.

### 5.6. Using an Argumentation Protocol to Analyze Negotiations

**Figure 5:**
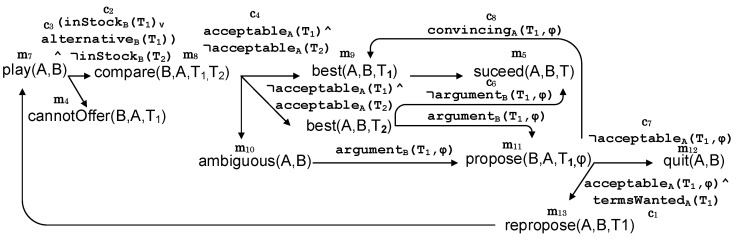
A simple argumentation protocol model for a game online. Additional (redundant) shorthand notation $c_{i}$/$m_{j}$ is introduced indicating different constraints and messages, respectively.

This section illustrates the strategies for the combination of protocols explained in Section 4. The protocol to analyze or $p_{1}$ is shown in Figure 1. Consider also Figure 5 as the $p_{2}$ protocol, the auxiliary protocol, which is used by agents (or developers) to improve protocol models of $p_{1}$. This second protocol shows a simple process of argumentation that a lessor could offer as a game online in popular social networks to find out customer preferences. In this vein, a number of smart city services are supported by social networks’ data [42]. This protocol for the online game is detailed below.

*A* starts the game with a message “play”. *B* proposes to compare two products, the first one being a product in stock or a possible alternative to be offered by this agent and the second one a product that is not currently available in stock. *A* answers with a message “best”, if a product proposed is considered better, or “ambiguous”, if they are considered equally acceptable. If the product selected is the first one, the protocol ends with a “succeed” message. If the product selected is the second one (which cannot be offered by *B*), *B* produces an argument supporting the first product with a message “propose”. Arguments are threats or prizes to make customers change their minds. If this argument convinces *A*, the protocol ends again with “succeed”. If the argument is not acceptable, *A* quits the game. Finally, if the argument is not convincing, but acceptable for the terms discussed, *A* proposes the kind of product that would be wanted with a message “repropose”, and the game starts again. Additional (redundant) shorthand notation $c_{i}$/$m_{j}$ is introduced continuing the numeration of $p_{1}$ in Figure 1.

Assume that we have 50 different customers and ten sellers who are able to offer products that satisfy the different mental state models used by customers. To add more difficulty, we implemented a number of seller agents who argue offering prizes or threats randomly regardless of the terms discussed. Runs were performed for both protocols with 50 customers and 10 sellers. The accuracy of a model of $p_{2}$ with the same learning strategies studied in Section 5.2 is shown in Table 3. Despite dealing with a protocol where learning is more complicated because the tuples include terms of two different products, an accuracy greater than 90% using a decision tree learning algorithm is obtained from a thousand conversations. If the random behavior of sellers is eliminated, this accuracy of the mental state model rises to 96%. Although these results are irrelevant to the use of data from $p_{2}$ to build context models of $p_{1}$, they give further evidence of the potential of the protocol analysis method presented in this paper.

As can be seen following the shorthand notation of the two protocols explained above, there are a number of common constraints ($c_{1}$, $c_{2}$, $c_{3}$, and $c_{4}$) and a series of messages that are used in both protocols ($m_{4}$ and $m_{5}$), which suggest that the combination of these protocols in a unified dataset is possible. To apply the first protocol combination strategy explained in Section 4, $p_{2}$ must include complete paths whose constraints allow paths of $p_{1}$ to be formed. Specifically, there are two paths meeting these requisites in $p_{2}$, both including a loop with the message “repropose” ($m_{12}$) and ending with “succeed” and “cannotOffer”, respectively.

$$\pi_{2.1}=\ldots \overset{c_{7}\left(T_{1}\right)\wedge c_{1}\left(T_{1}\right)}{\to }m_{13}\overset{}{\to }m_{7}\overset{\left(c_{2}\left(T_{1}\right)\vee c_{3}\left(T_{1}\right)\right)\wedge \neg c_{2}\left(T_{2}\right)}{\to }m_{8}\overset{c_{4}\left(T_{1}\right)\wedge \neg c_{4}\left(T_{2}\right)}{\to }m_{9}\overset{}{\to }m_{5}$$

$$\pi_{2.2}=\ldots \overset{c_{7}\left(T_{1}\right)\wedge c_{1}\left(T_{1}\right)}{\to }m_{13}\overset{}{\to }m_{7}\overset{\neg (\left(c_{2}\left(T_{1}\right)\vee c_{3}\left(T_{1}\right)\right)\wedge \neg c_{2}\left(T_{2}\right))}{\to }m_{4}$$

Note that $\pi_{2.1}$ and $\pi_{2.2}$ contain evaluations of the four constraints common to both protocols for some specific terms ($T_{1}$). Taking executions of these paths, the following three paths in the $p_{1}$ protocol can be derived:

$$\pi_{1.1}=\overset{c_{1}}{\to }m_{1}\overset{c_{2}}{\to }m_{2}\overset{}{\to }m_{5}$$

$$\pi_{1.2}=\overset{c_{1}}{\to }m_{1}\overset{\neg c_{2}\wedge c_{3}}{\to }m_{3}\overset{c_{4}}{\to }m_{5}$$

$$\pi_{1.3}=\overset{c_{1}}{\to }m_{1}\overset{\neg c_{2}\wedge \neg c_{3}}{\to }m_{4}$$

where $\pi_{2.1}$ allows $\pi_{1.1}$ and $\pi_{1.2}$ to be derived and $\pi_{2.2}$ allows $\pi_{1.3}$ to be derived. There are other paths that contain $\pi_{2.1}$ and $\pi_{2.2}$ in $p_{2}$ through more iterations in loops, but the complete paths that can be derived in $p_{1}$ from these longer paths are the same. For 1000 executions of $p_{2}$, only 27 of them were useful to derive a tuple to build the protocol model of $p_{1}$ by this method.

Following the third approach to the combination of protocols explained in Section 4, the logic consequence in the constraints of both protocols can be looked for. Specifically, it can be assumed that if a product proposed by a seller is acceptable to the customer, the customer ultimately also wants that product. That is, $acceptable_{A}\Rightarrow termsWanted_{A}$. Hence, if a path in the argumentation includes the former, we can assume the latter. Thus, a third path of $p_{2}$ can be added to deduce $p_{1}$’s information:

$$\pi_{2.3}=m_{7}\overset{\left(c_{2}\left(T_{1}\right)\vee c_{3}\left(T_{1}\right)\right)\wedge \neg c_{2}\left(T_{2}\right)}{\to }m_{8}\overset{c_{4}\left(T_{1}\right)\wedge \neg c_{4}\left(T_{2}\right)}{\to }m_{9}\overset{}{\to }m_{5}$$

**Figure 6:**
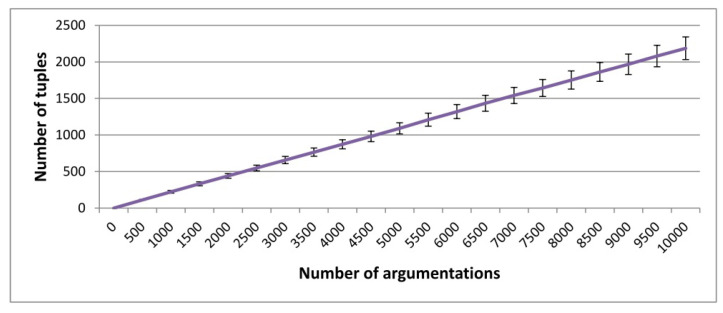
Average of tuples to improve a negotiation protocol model with argumentation executions across the total number of argumentations (100 experiments). Error bars show the standard deviation.

In this manner, tuples representing executions of $p_{1}$ that take paths $\pi_{1.1}$ and $\pi_{1.1}$ can be deduced. Although $\pi_{2.2}$ is very similar to this new path considered $\pi_{2.3}$, the fundamental difference is that the latter requires no loops, and it is the fastest and most direct way to the end of the argumentation protocol. After running 1000 conversations of the argumentation protocol, two-hundred nineteen tuples were extracted for the negotiation, i.e., almost 25% of the executions in $p_{2}$ were reused to study $p_{1}$. This significant increase shows the importance of flexible strategies in the combination of protocols to build hidden mental state models. The final result is that, whereas the accuracy of a model of $p_{1}$ for 1000 protocol executions was 94.30%, after including the 219 extracted tuples, this number rose to 97.45%. Figure 6 shows that for this specific implementation of the argumentation protocol shown in Figure 5, the number of argumentation tuples that can be used to study negotiation by improving its protocol model remains around 25% of the argumentation runs.

## 6. Conclusions and Future Work

In a smart city, we cannot assume that the implementation of all the devices involved is known, not even their manufacturers or operating systems. This situation also occurs in multi-agent systems, and therefore, they are a widely studied technology for the design of ambient intelligence. This opacity in agents’ mental states may hinder intelligent services from obtaining information from these. This paper proposes the use of semantic annotations in interaction protocols for multi-agent systems to allow machine learning methods to get insights into these mental states, even when the information comes from different protocols with distinct purposes in the smart city.

This proposed approach consists of obtaining theories, called hidden mental state models or protocol models, which can explain the behavior or decision-making of agents in the interaction protocol. Moreover, these models leverage the specific semantics exchanged in that conversation. The typical quantitative analysis, very present in the specialized literature, is capable of extrapolating models based on the number of successes in the execution of a protocol. However, more powerfully, the use of the presented machine learning models allows developers (and agents) to figure out under which semantic conditions these successes happened and to make predictions about how to improve such interactions.

Of course, a semantic analysis involves more difficulties than a quantitative approach. To succeed, designing interaction protocols properly is crucial, i.e., including sufficient semantic annotations. It is also necessary to deal with a series of problems that mostly have to do with how data are extracted from interaction protocols to form the training data. Some of these problems, for which this paper provides specific strategies, are: the presence in protocols of multiple agents, loops, paths of different lengths, different forms of termination, etcetera. Finally, the most significant contribution of this paper is that improving the learning of agents’ behaviors for a specific interaction model through experiences of different communication protocols is feasible. The underlying idea is that if semantic annotations are shared (or related by the logic consequence), learning from them is possible regardless of the specific protocol or purpose for which they are used.

This paper shows experimental results for a car renting multi-agent system. The explained methods are applied: to feed general machine learning algorithms with past interactions in the system; to learn a specific hidden mental state in an agent; to predict the outcome of the negotiations; to study incoherent behaviors and changes of preferences in agents’ mental states; and to use information from other protocols, an online game in the case study, to improve the learned models for the car renting system. Although our approach is agnostic to the machine learning paradigm, we experimented with five algorithms of different learning paradigms. Deep learning techniques such as the multi-layer perceptron outperform other methods in accuracy. However, the interpretability of the model is lost, and its training has a computational cost several orders of magnitude higher than the alternatives studied.

Regarding our future work, the most immediate is the application of the ideas presented in this paper to more complex and realistic scenarios for smart cities such as: transport services [43], Ambient Assisted Living (AAL) services [44], or e-commerce systems (Amazon, eBay, etcetera). Another important future work is implementing our formal framework in a multi-agent system platform. In this line, the use of annotations in Jason is an extremely flexible mechanism frequently used to extend AgentSpeak (http://jason.sourceforge.net/doc/tech/annotations.html). Furthermore, experiments with more advanced machine mining techniques are also necessary such as Recurrent Neural Networks (RNN). These networks are able to remember a given sequence of messages to conduct a classification without requiring the same length vector to represent a protocol execution. Finally, exploring the interpretability and explainability of the machine learning models used is essential. As shown in a recent review [45], most prediction and decision-making methods in intelligent environments are interpretable. Therefore, the use of grey-box models, which combine the interpretability of a white-box model with the accuracy of a black-box model, will be considered [40].

## Figures and Tables

**Table 1:** Time in seconds required to build protocol outcome models in a laptop Dell xps 13 9343 (Core i5 5200U, 8 GB RAM). Learning algorithms: a decision tree algorithm (J48), a rule induction method (NNge), a Bayesian network classifier (BayesNet), a feed-forward artificial neural network (multilayer perceptron), and a clustering-based classification (SimpleKMeans).

Negotiations	J48	NNge	Bayes	Perceptron	Clustering
50	0.04	0.006	0.002	0.915	0.007
100	0.04	0.002	0.002	1748	0.002
250	0.001	0.013	0.002	4366	0.005
500	0.001	0.034	0.002	8737	0.008
750	0.003	0.063	0.002	13,035	0.012
1000	0.003	0.103	0.002	17,337	0.019
2000	0.006	0.36	0.002	35,048	0.042
3000	0.012	0.682	0.006	52,647	0.055
4000	0.024	1046	0.005	70,300	0.076
5000	0.019	1468	0.002	88,219	0.083

**Table 2:** Some contradictory negotiations with $C_{2}$. An additional “id” field has been added to refer to these examples in the text.

id	doors	persons	lug_boot	safety	buying	maint	outcome
1	2	2	small	high	?	?	F
2	2	2	small	high	low	low	S
3	3	4	med	high	?	?	F
4	3	4	med	high	low	low	S
5	5-more	more	big	low	med	med	N
6	5-more	more	big	low	med	med	S
7	5-more	more	med	high	low	low	N
8	5-more	more	med	high	low	low	S

**Table 3:** Accuracy in cross-validation for the protocol model obtained from interactions using the argumentation protocol.

Conversations	With Randomness	Without Randomness
1000	90%	96%
5000	95%	99%
10,000	95%	99%
